# EGFR p.V774M/p.L833V compound mutations in lung adenocarcinoma responded well to almonertinib: a case report

**DOI:** 10.3389/fonc.2023.1159308

**Published:** 2023-05-12

**Authors:** Hai Pan, Linlin Zhang, Fanlu Meng, Shasha Guan, Diansheng Zhong

**Affiliations:** Department of Medical Oncology, Tianjin Medical University General Hospital, Tianjin, China

**Keywords:** lung adenocarcinoma, EGFR, almonertinib, compound mutation, V774M/L833V

## Abstract

**Background:**

There are about 10-15% of uncommon EGFR mutations found in NSCLC patients, and their sensitivity to EGFR TKIs still lack sufficient clinical evidence, especially for rare compound mutations. Almonertinib is the third generation of EGFR-TKI that has demonstrated excellent efficacy in classical mutations, however, effects in rare mutations have also been rarely reported.

**Case presentation:**

In this case report, we present a patient with advanced lung adenocarcinoma with a rare EGFR p.V774M/p.L833V compound mutations, who achieved long-lasting and stable disease control after first-line Almonertinib targeted therapy. This case report could provide more information for therapeutic strategy selecting of NSCLC patients harboring rare EGFR mutations.

**Conclusion:**

We report for the first time the long-lasting and stable disease control with Almonertinib for EGFR p.V774M/p.L833V compound mutations treatment, hoping to provide more clinical case references for the treatment of rare compound mutations.

## Introduction

Lung adenocarcinoma is the most common type of non-small cell lung cancer, and conventional chemotherapy-based therapy has failed to significantly improve the prognosis of advanced patients. Targeted therapies based on driver genes significantly prolong patient survival. EGFR is the most common driver gene, and the most predominant mutant forms are EGFR p.L858R and 19del, which are called classical mutations.

With the extensive research and widespread use of detection tools such as next-generation sequencing and liquid biopsy in the area of oncology, more and more rare EGFR mutations have been identified in patients with advanced lung cancer (1, 2). Chinese data revealed that approximately 11.9% of EGFR mutations in lung cancer are rare mutations, mainly occurring in different types of genomic alterations within exon18-21, including the well-known mutations exon20ins, exon18 G719X, exon20 S768I, and exon21 L861Q, as well as some more rare mutations (3). The major EGFR TKIs currently used in clinical practice lack targeted clinical studies and major guidelines do not have clear dosing recommendations, especially for patients with those rare compound mutations, due to these patient bases being so small and varied, except a few studies of afatinib enrolling a small number of patients with rare mutations.

The common EGFR exon20 mutation is the EGFR p.T790M secondary mutation following first-generation TKI resistance, which is a change from a smaller threonine to a larger methionine in the side chain. As a result, this alteration affects the binding of the TKIs to the drug binding pocket. Although afatinib can form covalent binding at position C797, its chemical structure will still be affected by the spatial site block of T790M, while osimertinib can not only form covalent binding, but also its indole ring is arranged in parallel with the mutated T790M, so it well avoids the spatial site block effect. Therefore, we speculate that mutations located at the end of exon 20 may respond better to third-generation TKIs than afatinib.

Here, we provide a case of a patient harboring EGFR p.V774M/p.L833V compound mutations with good efficacy under Almonertinib-targeted therapy to accumulate more clinical evidence for the treatment of rare compound mutations.

## Case description

**Figure 1:**
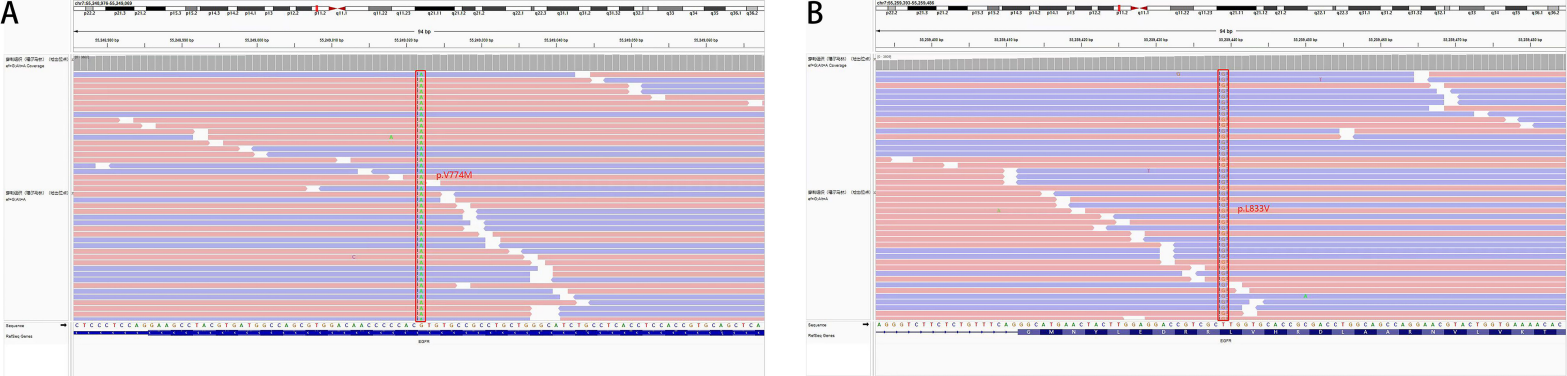
Tissue biopsy next-generation sequencing showed **(A)** EGFR p.V774M mutation in exon 20 and **(B)** EGFR p.L833V mutation in exon 21.

**Figure 2:**
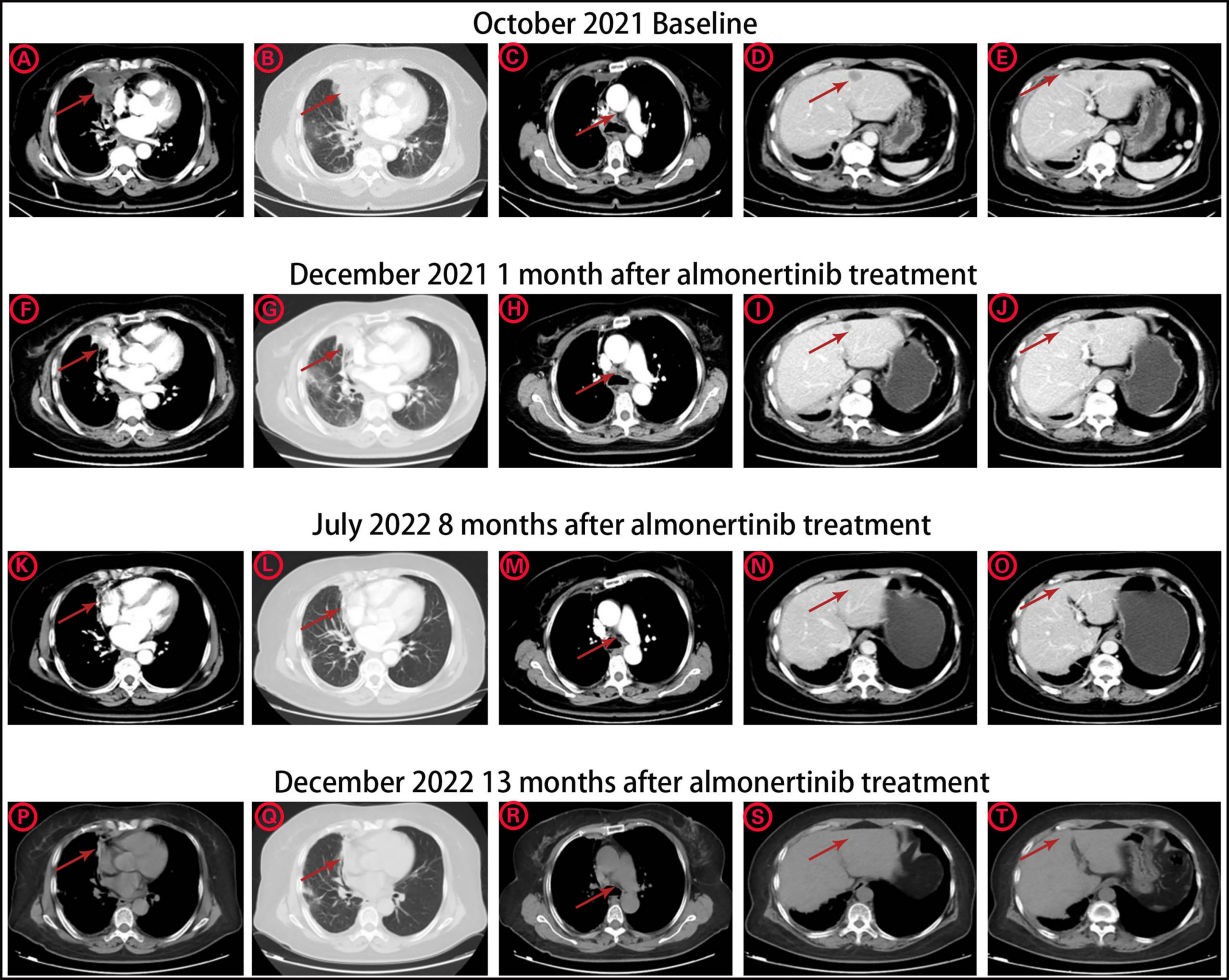
The CT image changes of pulmonary primary tumor site, mediastinal lymph node metastasis, and liver metastases **(A-E)** before Almonertinib treatment in October 2021. **(F-J)** 1 month after Almonertinib treatment in December 2021. **(K-O)** 8 months after Almonertinib treatment in July 2022. **(P-T)** 13 months after Almonertinib treatment in December 2022.

The patient was a 63-year-old female patient with no smoking history, who was found to have right pleural effusion and right middle lobe mass on chest CT examination in September 2021 due to chest tightness and shortness of breath, and a subsequent PET-CT examination revealed a right middle lobe malignant tumor with right hilar, mediastinal lymph node, pleural, and liver metastases. The pathology of the lymph node biopsy under the ramus of tracheoscopy confirmed adenocarcinoma of the lung, and the baseline stage was T2aN2M1c stage IV. Then, tissue and blood ctDNA next-generation sequencing was performed(423-gene panel, Genecast Biotechnology Co., Ltd.), suggesting an EGFR p.V774M/p.L833V compound mutations, accompanied by TP53 p.R248W and PIK3CA p.C420R mutations. Among them, EGFR p.V774M and EGFR p.L833V mutations are located in exon20 and exon21 respectively (**Figure 1**). Starting in November 2021, oral Almonertinib 110 mg QD targeted therapy was administered. After one month of treatment, the lung mass, lymph nodes, and liver metastases were found to be smaller than before, and the pleural fluid was also reduced (**Figure 2**). This response was evaluated as partial remission (PR), and when blood ctDNA next-generation sequencing was conducted again, it was discovered that the EGFR mutation had disappeared, indicating that the treatment was successful. Throughout that time, the tumor was under control. In July 2022, the ctDNA next-generation sequencing was sent again dynamically, and there was still no EGFR mutation, only ARID2 p.M1288I mutation (**Table 1**). During treatment, no major TKI-related adverse events were found. As of the follow-up cut-off in December 2022, the patient’s disease was stable and Almonertinib remained consistently beneficial with a PFS of more than 13 months. It is worth mentioning that no metastases were found on enhanced MRI scans of the head during the initial treatment and follow-up after targeted therapy.

**Table 1:** Dynamic liquid biopsy genomic changes during treatment.

Gene	Amino acid change	Nucleotide change	Baseline	2months after Almonertinib	8months after Almonertinib
EGFR	c.2320G> A	p.V774M	2.2%	–	–
EGFR	c.2497T>G	p.L833V	2.55%	–	–
ERBB4	c.2005C>G	p.L669V	0.65%	–	–
KDR	c.159C>A	p.C53*	0.47%	–	–
MDM2	c.166A>G	p.M56V	1.75%	–	–
MITF	c.28G>T	p.V10F	2.44%	–	–
PIK3CA	c.1258T>C	p.C420R	1.09%	–	–
TP53	c.742C>T	p.R248W	1.4%		–
CTCF	c. 1207 + 1G>A	–	–	0.43%	–
ARID2	c.3864G>A	p.M1288I	–	–	0.43%

"-" means that no mutation was detected.

## Discussion

TKIs targeting classical EGFR mutations has become the standard first-line treatment for advanced NSCLC, and all three generations of EGFR TKIs have achieved substantial clinical benefits. However, studies on uncommon point mutations have concentrated on the high-incidence variants G719X, S768I, and L861Q. According to the LUX-LUNG2/3/6 meta-study (4), afatinib showed greater overall efficacy than first-generation TKIs, with an mPFS of 10.7 months and an ORR of 71% for these point mutations. A KCSG-LU15-09 study in Korea (5)investigated the efficacy of the third-generation TKI osimertinib in rare point mutations and showed an ORR of 50% and an mPFS of 8.2 months, also showing a good clinical benefit. Additionally, another study of osimertinib in rare mutations (6) also demonstrated similar clinical efficacy, with an ORR of 63% and PFS of 5.5 months in the first-line treatment subgroup of the study, and better efficacy of 13.7 months in the G719X mutation group, while 81% of patients showed ctDNA reduction after osimertinib treatment, suggesting effective treatment. Therefore, for the treatment of uncommon mutations, afatinib or osimertinib should be given priority. Compound mutations are common among rare mutations, and it has been noted that patients with compound mutations have shorter OS than those with single mutation (7). The treatment of compound mutations has also only been reported in the literature in a small number of cases. A preclinical analysis by Japanese scholars with large-scale high-throughput sequencing evaluated the sensitivity of 69 different EGFR mutation combinations in cell lines to various generations of TKI and showed that 62 were highly sensitive to afatinib, and 45 were highly sensitive to osimertinib, especially the compound mutations combined with EGFR p.T790M mutations (8). As a result, it is noted that second or third-generation EGFR TKIs are more suited to treat EGFR compound mutations. Almonertinib is also a third-generation TKI, but compared to osimertinib, it only replaces the original methyl group with a cyclopropyl group in the indole ring, and this change does not affect the spatial location of protein binding. Therefore, Almonertinib should theoretically have some efficacy for rare mutations as well.

The two mutation sites, in this case, are EGFR p.V774M and p.L833V, and EGFR p.L833V is the most frequently reported mutation. EGFR p.L833V seldom occurs alone and is often paired with other unusual mutations, the most prevalent of which is the EGFR p.L833V/H835L compound mutations. Similar to the classical mutations of EGFR p.L858R and exon19del, the EGFR p.H833V mutation is located in exon 21, which is far from the ATP binding pocket. It has less of an influence on the TKI and ATP binding site and is therefore theoretically effective for all generations of EGFR TKIs. Reviewing the previous relevant literature, Yang et al (9) described a case of advanced lung adenocarcinoma with the EGFR p.L833V/H835L compound mutations treated with second-line gefitinib and had a PFS of 8.5 months. Long et al (10), on the other hand, identified a case of EGFR p.L833V/H835L mutations from blood and pleural fluid ctDNA and achieved a PFS of more than 10 months with first-line afatinib treatment. In addition, Zhou et al (11) present a case of EGFR p.L833V combined with the EGFR p.G719A mutation, which also had good efficacy with the icotinib, with 8 months PFS. In addition to the double mutation form of EGFR p.L833V, more rare triple mutation combinations also occurred. The EGFR p.L833V/H835L/E709K triple mutation was observed in an Italian patient, and afatinib treatment also contributed to a PFS of more than 2 months (12). Another patient with a triple mutations was an EGFR p.L833V/H835L/R670W combinations, who was resistant to the first-line gefitinib and achieved clearance of the EGFR p.L833V/H835L/R670W mutation with third-line afatinib treatment, with a PFS of more than 7 months, followed by a re-treatment blood test due to progression of brain metastases with the EGFR p.L833V/H835L/T790M compound mutations, with the same remarkable effect of fifth-line osimertinib treatment (13). Moreover, data from another large European sequencing briefly reported three cases of EGFR p.L833V compound mutations, two EGFR p.L883V/H835L, and one EGFR p.L833V/G719A, only one of which responded well to erlotinib in combination with chemotherapy and the other two with poor efficacy with first generation TKI (14) (**Table 2**). Therefore, the locus EGFR p.L833V is a sensitive mutation site for all generations of EGFR TKI based on the structural change and clinical cases.

**Table 2:** EGFR p.L833V mutation targeted therapy case summary.

Author	Sex	Year	Smoking	Race	Mutation	Treatment	Line	Efficacy	PFS
Yang et al	M	89	Mild	Asian	L833V/H835L	Gefitinib	2	PR	8.5m
Long et al	M	65	No	Asian	L833V/H835L	Afatinib	1	PR	10+m
Zhou et al	F	74	No	Asian	L833V/G719A	Icotinib	1	PR	8m
Sousa et al Case2	NA	NA	NA	Euro	L833V/H835L	Erlotinib +Chemo- therapy	1	SD	9m
Sousa et al Case13	NA	NA	NA	Euro	L833V/G719A	Gefitinib	1	PD	2m
Sousa et al Case33	NA	NA	NA	Euro	L833V/H835L	Erlotinib +Bev	1	PD	NA
Frega et al	M	70	Yes	Euro	L833V/H835L/E709K	Afatinib	1	PR	2+m
Qin et al	M	36	No	Asian	L833V/H835L/R670W	Gefitinib	1	PD	1m
					L833V/H835L/R670W	Afatinib	3	PR	7+m
					L833V/H835L/T790M	Osimertinib	5	PR	3+m
This	F	63	No	Asian	L833V/V774M	Almonertinb	1	PR	13+m

M, male; F, female; Euro, European; PR, partial remission; SD, stable disease.

PD, progressive disease; m, month; NA, not available.

In contrast, the mutation site, p.V774M of EGFR exon20, is located on the C-terminal inner surface of the αC helix of the EGFR molecule, a common region in exon20ins mutation, the posterior loop region of the α helix, which is also the binding region for ATP and EGFR TKI. A recent large study (15) evaluated mutations in 16715 NSCLC patients with EGFR mutations, established the relationship between drug sensitivity and mutation structure, and classified EGFR mutations into four distinct subgroups. According to this classification, EGFR p.V774M belongs to the P-loop and α-C helix compression type, or PACC type, a class of mutations that compresses the volume of the ATP binding pocket and is predicted to be less effective against osimertinib. But this mutation has rarely been reported in real treatment cases. Yang et al (16) reported a case of a patient with the EGFR p.H733L/V774M compound mutations in whom little effect was achieved with gefitinib, while 12 months of tumor control was achieved with osimertinib in combination with bevacizumab. Another patient with the EGFR p.H773L/V774M compound mutations showed a poor outcome with afatinib treatment (17). Then there was a large retrospective study in France where there were two cases of EGFR p.H773L/V774M mutation as well as one case of EGFR p.V774M single mutation, all of which had a poor response to first-generation TKIs (**Table 3**).

**Table 3:** EGFR p.V774M mutation targeted therapy case summary.

Author	Sex	Year	Smoking	Race	Mutation	Treatment	Line	Efficacy	PFS
Yang et al	M	50	Yes	Asian	H773L/V774M	Osimertinib	3	PR	12m
Chen et al	F	56	No	Asian	H773L/V774M	Afatinib	1	PD	NA
This	F	63	No	Asian	V774M/L833V	Almonertinib	1	PR	13+m

M, male; F, female; PR, partial remission; PD, progressive disease; m, month; NA, not available.

Therefore, in our case, for EGFR p.L833V/p.V774M, the first double mutations identified, EGFR p.L833V is theoretically sensitive to all generations of TKI, while the EGFR p.V774M mutation, although structurally predicted to be perhaps less efficacious for third-generation TKI, showed significant efficacy in real cases and poor efficacy of afatinib has been reported, so we choose to treat with Almonertinib. Fortunately, the patient not only demonstrated shrinkage of target lesions as well as metastases and stable control of pleural fluid after treatment but also achieved clearance of EGFR mutant sites during subsequent multiple simultaneous ctDNA monitoring. Clearance of ctDNA at the first post-treatment assessment has been reported to be associated with higher ORR and longer survival (18, 19), as confirmed by a PFS of more than 13 months. The mechanism behind this may be related to the EGFR p.L833V/V774M co-mutation, resulting in a more structurally complex change different from the single mutation, but this still requires further studies to follow.

In conclusion, we report for the first time the long-lasting and stable disease control with Almonertinib for EGFR p.V774M/p.L833V compound mutations treatment, hoping to provide more clinical case references for the treatment of rare compound mutations.

## Data availability statement

The original contributions presented in the study are included in the article/supplementary material. Further inquiries can be directed to the corresponding author.

## Ethics statement

Written informed consent was obtained from the individual(s) for the publication of any potentially identifiable images or data included in this article.

## Author contributions

FM and SG provided the clinical information of this patient. HP and LZ wrote the manuscript. DZ rigorous reviewed and gave financial support. All authors contributed to the article and approved the submitted version.
